# Effect of Flanking Sounds on the Auditory Continuity Illusion

**DOI:** 10.1371/journal.pone.0051969

**Published:** 2012-12-14

**Authors:** Maori Kobayashi, Makio Kashino

**Affiliations:** 1 Department of Science and Technology, Meiji University, Kawasaki, Kanagawa, Japan; 2 NTT Communication Science Laboratories, NTT Corporation, Atsugi, Kanagawa, Japan; 3 Interdisciplinary Graduate School of Science and Engineering, Tokyo Institute of Technology, Yokohama, Kanagawa, Japan; University of Leicester, United Kingdom

## Abstract

**Background:**

The auditory continuity illusion or the perceptual restoration of a target sound briefly interrupted by an extraneous sound has been shown to depend on masking. However, little is known about factors other than masking.

**Methodology/Principal Findings:**

We examined whether a sequence of flanking transient sounds affects the apparent continuity of a target tone alternated with a bandpass noise at regular intervals. The flanking sounds significantly increased the limit of perceiving apparent continuity in terms of the maximum target level at a fixed noise level, irrespective of the frequency separation between the target and flanking sounds: the flanking sounds enhanced the continuity illusion. This effect was dependent on the temporal relationship between the flanking sounds and noise bursts.

**Conclusions/Significance:**

The spectrotemporal characteristics of the enhancement effect suggest that a mechanism to compensate for exogenous attentional distraction may contribute to the continuity illusion.

## Introduction

In daily situations, multiple acoustic events often overlap in time, creating a complex auditory scene. Listening to a target sound against extraneous sounds is a challenge for auditory information processing, because parts of the target sound may be masked by extraneous sounds and become unavailable for recognizing the target sound. However, the human auditory system has a remarkable ability to compensate for such temporal masking. A compelling demonstration of this ability is the auditory continuity illusion, by virtue of which a target sound (the inducee) which is momentarily interrupted by an extraneous sound (the inducer), is perceived to be continuous (i.e., it continues to be heard through the inducer), even if the inducee is physically absent during the presence of the inducer [1], [2].

Previous studies have examined some limiting factors to the perception of this phenomenon [1]–[5], [7]. For example, it does not occur if the inducee and the inducer are separated from each other by a short pause [3], or have nonabrupt transitions [4]. Also, this illusion is reduced when the interaural phase difference of the inducer and that of the inducee are different [5]. Further, a close relationship between the continuity illusion and masking has been well established. Indeed, Warren et al. [2] manipulated the level of the inducers and the frequency of the inducees and measured the continuity limits (i.e., the highest level of the inducees at which they were heard as continuous). They found that the frequency-dependent continuity limit curve resembled the curve they obtained when the inducer was left on continuously, and the masked detection threshold of the pulsed inducee was determined [2].

This masking potential rule makes sense from an ecological perspective, as the perceptual restoration of a missing part would be valid provided the part would have been masked by another sound. The extent of masking depends on the difference in frequency and sound pressure level between the masker and target sounds, reflecting the frequency selectivity of the auditory filters, that is, a bank of band-pass filters at an early stage of auditory processing [7]. It has therefore been suggested that the auditory filters play a crucial role in the continuity illusion [6]. Moreover, in order for the continuity illusion to occur, the neural activity evoked by the higher amplitude inducer must include the neural activity corresponding to the continuation of the lower amplitude inducee.

The involvement of processes beyond the auditory filters, or more specifically, the role of attention in the continuity illusion is unclear. It has been shown that the continuity illusion is automatic and compulsory under appropriate condition [8], [9], and does not require the focus of attention [10]. These findings, together with the masking potential rule, suggest that the neural representation corresponding to the illusory continuity is created at relatively early stages of auditory information processing, independent from the influence of attention. For instance, Drake and McAdams [11] showed the instruction have no effect in the continuity illusion. Also, our recent study indicates that a transient visual stimulus, synchronized with the onset of the noise inducer, enhances the apparent continuity of the tone inducee [12]. This visual effect cannot be readily explained by the masking potential rule, suggesting the involvement of a processing stage higher than the auditory filters, such as crossmodal binding and attention.

To further clarify the role of attention in the continuity illusion, we have examined whether a sequence of flanking transient sounds (the flanker) affects the apparent continuity of a tone inducee repeatedly alternated with a bandpass noise inducer with various frequency differences (Experiment 1) and different temporal relationships (Experiment 2) between the inducer and flanker. The extent of apparent continuity was measured as the continuity limit, which was defined as the highest inducee level at which a listener judged the inducee as continuous. The higher the continuity limit, the stronger is the continuity illusion. If the effect of the flanker is not restricted to the regions spectrotemporally adjacent to the inducer, this would indicate the involvement of processes beyond the auditory filters, possibly stimulus-driven (i.e., exogenous) attention.

## Materials and Methods

### Ethical Statement

Informed written consent was obtained from each participant before the experiments were conducted. All procedures were approved by the ethics committee of the NTT Communication Science Laboratories.

### Participants

Seven young adults (20–29 years of age) participated in this study. All participants had normal hearing (thresholds of 15 dB HL or better over the range 125–8000 Hz), and were naïve to the purpose of the experiments.

### Stimuli and Apparatus

The tests were conducted in a soundproof room. The stimuli were produced digitally on a computer (Apple Power Mac G5) and presented through a D/A converter (STAX DAC-TALENT), a driver unit (STAX SRX-1), and electrostatic headphones (STAX SR-PRO) to both ears of the participants. The experimental sequence was controlled using MATLAB 7.2 (Mathworks) with a Psychophysics toolbox extension.

**Figure 1:**
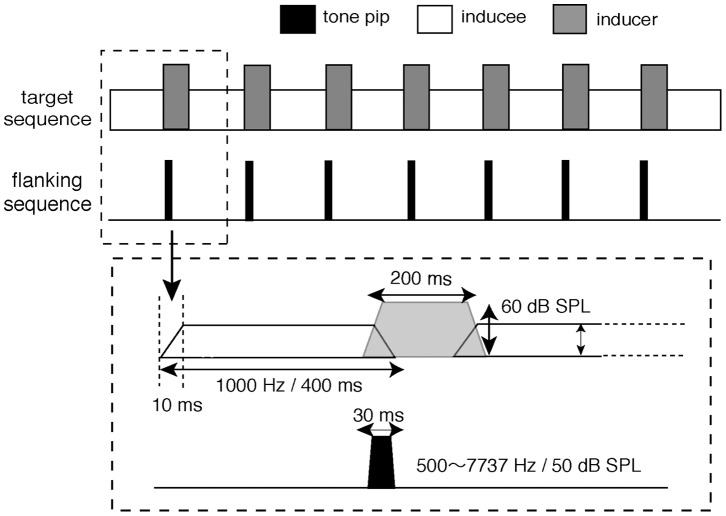
Stimuli used in Experiment 1. The stimuli employed for measuring the continuity limit consisted of a 500-Hz sinusoidal inducee alternating with a 1000-Hz, one-third-octave noise band inducer. The flanking sequence consisted of seven tone pips.

The target sequence consisted of two sounds that were presented in alternation, as shown in Figure 1. The inducee was a 400-ms, 1000-Hz sinusoidal tone, and the inducer was a 200-ms, one-third-octave noise band centred at 1000 Hz. To reduce switching transients, both the inducer and inducee were gated on and off with a 10-ms raised-cosine ramp, with overlapping adjacent ramps. The inducer and inducee were alternated 7 times so that the entire sequence lasted 4450 ms. The inducer was maintained at 60 dB SPL, while the inducee level was varied, depending on each listener’s response (see Procedure). The flanker consisted of seven sinusoidal tone pips whose duration was 30 ms, including the 10-ms raised-cosine ramps. The tone pips were always presented at 50 dB SPL. The frequency and timing of the tone pips in the flanker varied across conditions.

### Procedure

The participants judged whether the inducee was perceived to be continuous in each trial. The continuity limits were measured using the method of limits. In the first trial of an ascending series of judgments, the initial level of the inducee level was randomly chosen from within the range 42 to 45 dB SPL, and the level was increased in steps of 3 dB at regular intervals in successive trials until the listener reported discontinuity in the inducee. For a descending series of trials, the initial level of the inducee was randomly chosen from within the range 66 to 69 dB SPL, and the level was decreased in 3 dB steps until the listener judged the inducee to be continuous. For each condition, the continuity measurement contained 12 series (6 ascending and 6 descending). The mean of all 12 termination points was taken as the continuity limit for the condition. The order of the conditions was randomized for each participant.

### Conditions

Experiment 1 was designed to examine the effect of frequency separation between the flanker and inducee. Five conditions were tested: four flanker frequencies (500, 2073, 4040, and 7737 Hz) and a reference condition in which no flanker was presented. The onset of the flanker was synchronized with the onset of the inducer: the SOA between the flankers and the inducers was 0 ms. The participants took part in a practice session, which was followed by experimental sessions. The practice session lasted about 5 min, and the experiment lasted about 60 min, including rest time.

Experiment 2 was designed to examine the effect of the temporal relationship between the flanker and inducer; seven conditions were tested–five stimulus onset asynchronies (SOAs) between the flanker and inducer (–200, –100, 0, +100, and +200 ms; negative values indicate that the inducer preceded the flanker and positive values indicate the opposite), a random condition, and a reference condition. In the random condition, the SOA between each tone pip in the flanker and each noise burst in the inducer was randomly chosen from seven values between –396 and +396 ms, such that the temporal pattern of the tone pips in the flanker was not correlated with that of the noise bursts in the inducer. In the reference condition, no flanker was presented. The frequency of the flanking tone pips was fixed at 5000 Hz. The participants took part in a practice session, which was followed by experimental sessions. The practice session lasted about 5 min, and the experiment lasted about 90 min, including rest time.

## Results

### Experiment 1

**Figure 2:**
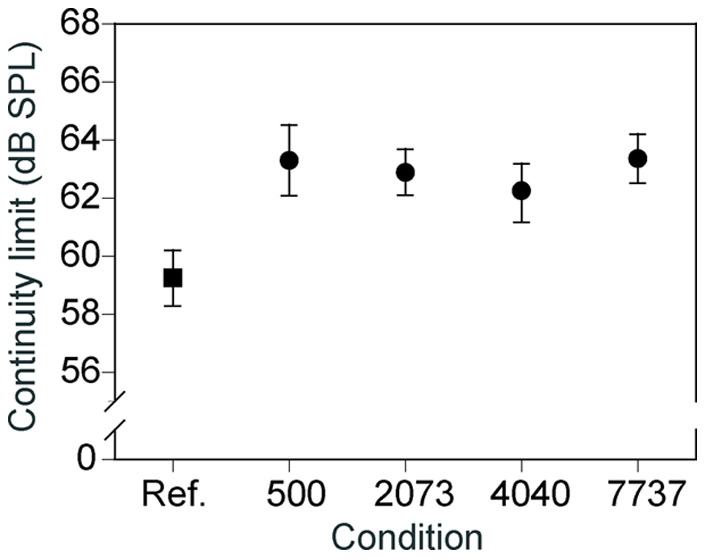
Continuity limits for each of the five conditions in Experiment 1 (Ref, the reference condition; the numbers are the frequencies of the tone pip in Hz).

Figure 2 shows the mean continuity limits across the seven listeners. A repeated-measures ANOVA on the continuity limits, revealed a significant effect of condition [*F* (4,34) = 4.08, *p*<0.05]. Pairwise comparisons, conducted using the Tukey HSD test, indicated that all conditions with flankers differed from the reference condition (*p*<0.05). On the other hand, there was no difference between the 500-Hz and the other frequency conditions.

These results indicate that the continuity illusion was enhanced by the flanker. Moreover, the magnitudes of the flanker effects were not different among the 4 frequency conditions, even when the frequency was more than that of the inducee by 3 octaves.

### Experiment 2

**Figure 3:**
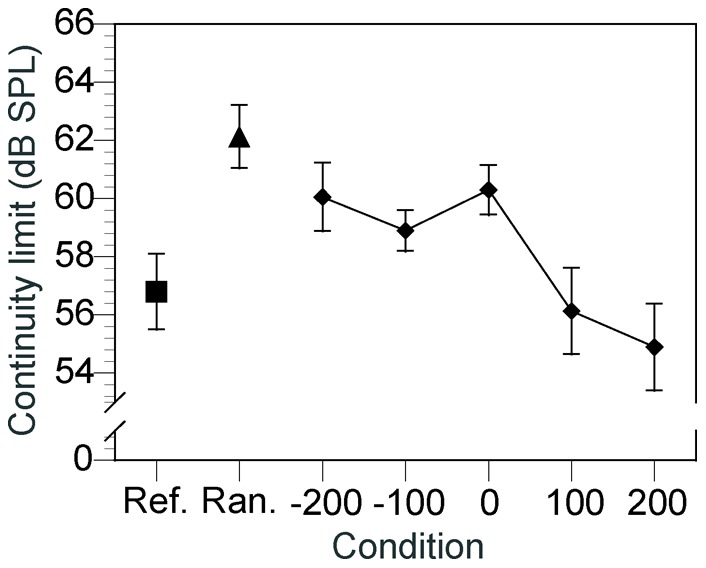
Continuity limits for each of the seven conditions in Experiment 2 (Ref, the reference condition; Ran, the random condition; the numbers are the stimulus onset asynchrony values in milliseconds). Negative values indicate that the tone pip preceded the inducer and positive values indicated that the inducer preceded the tone pip.

Figure 3 shows the mean continuity limits across the seven listeners. A repeated-measures ANOVA on the continuity limits revealed a significant effect of condition [*F*(6,48) = 11.63, *p*<0.01]. Post-hoc comparisons, revealed significant differences between the reference condition and the −200, 0, and random conditions [reference vs. −200 ms, 0 ms: *p*<0.05; reference vs. random: *p*<0.01]; between the random condition and the −100, +100, +200-ms conditions [random vs. −100-ms: *p*<0.05; random vs. 100-ms, 200-ms condition: *p*<0.01]; between the 0 and the 100, 200-ms conditions [*p*<0.01]; between the –200-ms and 100, 200-ms condition [*p*<0.05]; and between the –100 and the 200-ms conditions [*p*<0.01].

These results confirm the finding of Experiment 1 that the flanker synchronized with the onsets of the inducer enhanced the continuity illusion. Moreover, the magnitude of the enhancement effect depended on the temporal relationship between the inducer and flanker. A significant effect was also observed for the random condition in which the onset timing of tone pips in the flanker was not correlated with that of noise bursts in the inducer. On the other hand, when the flanker was presented after the inducer, it had no significant effect on the illusory continuity. Also, there were no significant difference between the reference and –100-ms condition.

## Discussion

In this study, we examined the effect of a sequence of flanking transient sounds (tone pips) on the apparent continuity of a tone inducee repeatedly alternated with a bandpass noise inducer at regular intervals. We found that when the flanker was synchronized with the inducer, the apparent continuity was significantly enhanced irrespective of the frequency separation between the inducee and flanker. Moreover, the flanker effect depended on the temporal relationship between the flanker and inducer. A particularly large enhancement was observed when the flanker was synchronized with the inducer and preceded it by 200 ms, or was presented at a random time relative to the inducer.

These results cannot be readily explained by the masking potential rule. Auditory masking is strongly influenced by frequency selectivity, which reflects the tuning of the auditory filters, and timing, having maximum effect when the masker and the target are presented simultaneously. According to the masking potential rule, the flanker with a frequency different from that of the inducer should not influence the illusion. However, the present study showed that the continuity illusion was influenced by the flanker, even when the flanker frequency was more than that of the inducer by 3 octaves. These results show that the effect of the flanker on the continuity illusion observed in this study is clearly different from the spectrotemporal tuning of masking. Therefore, it is unlikely that the enhanced effect is due to an increase in masking potential. It is suggested that a process that covers wide frequency regions, well beyond the bandwidth of a single auditory filter, is involved in the generation of apparent continuity.

What mechanism then causes the flanker to enhance illusory continuity? One possible mechanism is stimulus-driven (i.e., exogenous) attention. To efficiently process a vast amount of information in an ever-changing, complex auditory scene, the human auditory system has the capability of dynamically assigning processing resources or attention according to sensory input [10]. A salient change in the sensory input caused by the onset of a new acoustic event would require more processing resources than a steady input produced by an ongoing event, as the former is less predictable with respect to the preceding information. As a consequence, fewer processing resources would be left for the stationary, ongoing event. Nevertheless, the representation of the ongoing event should be maintained to achieve a stable interpretation of the auditory scene. We propose that the human auditory system has a mechanism that compensates for the disruption of attention caused by demanding inputs, and this compensation mechanism contributes to the continuity illusion in addition to the mechanism that compensates for the energetic masking that results from competition between the target and the masker at the periphery of the auditory system, that is, overlapping excitation patterns in the cochlea or auditory nerve. According to this view, the present results can be explained as follows: The transient sounds in the flanker, together with the sudden onsets of noise bursts in the inducer, capture attention that would otherwise have been directed to the inducee. Then, a compensation for attentional disruption takes place, resulting in enhanced apparent continuity for the inducee: when attention is engaged in another event, the occurrence of the gap in the inducee may go undetected and the past auditory scene may be maintained. This explanation is consistent with the temporal dependence of the flanker effect found in the present study, taking into account previous findings that attentional disruption is larger when a distracter is presented before a target than after a target, as shown by, attentional blink [13], [14] and attentional capture [15]. Attentional blink refers to the phenomenon whereby correct identification of the first target impairs the processing of the second target that is nearby in time in serial auditory/visual presentation. Interestingly, previous studies on attentional blink show that the phenomenon does not occur when the first target is presented 100 ms before the second target [13]. These findings are similar to our results demonstrating no effect under the −100-ms condition. The large effect found in the random condition also makes sense, as the timing of each tone pip in the flanker is unpredictable and would require considerable processing resources [16]. Further, the theory does not contradict the present findings that the flanker effects have little frequency selectivity. From these findings, the presentation of the flankers was more important than their frequency for the continuity illusion when the flanker was presented for a short time, such as this experiment.

The idea that attention is involved in the continuity illusion may seem contradictory to previous findings that the continuity illusion can occur automatically outside the focus of attention [10], but this is not necessarily so. The present results do not suggest that the continuity illusion requires focussed attention on the inducee. Rather, the continuity illusion can be enhanced when attention is taken *away* from the inducee. On the other hand, a more recent study using fMRI has suggested that the continuity illusion operates independently of attentional states [17]. While it is not clear why these differences between the fMRI study and our results exist, we speculate that the kinds of stimulus sequence and/or the strength of the continuity illusion may be possible explanations. The differences in the experimental conditions may have resulted in a difference of the magnitude of the attention effect on the continuity illusion.

Additionally, it should also be noted that exogenous attentional distraction alone is not sufficient to produce the continuity illusion. In the present study, the inducer exists and has spectrotemporal characteristics that may mask the inducee. We cannot deny that the masking potential rule is a necessary condition for the complete continuity illusion to occur; rather, exogenous attentional distraction has only a modulatory effect on the limit of the continuity illusion.

Our previous study reported a somewhat similar enhancement of the auditory continuity illusion by presenting a visual transient stimulus (flash) at the onset of the inducer noise [12]. These studies show that the transient signals have an enhancement effect on the continuity illusion. Although these effects are not exactly the same, we think that the present results are consistent with previous results. The visual effect was largest when the flash was synchronized with the onset of the inducer noise and decreased as the SOA between the two stimuli became larger in both directions. The flash had no significant effect on the auditory continuity illusion when the timing of the flash was random relative to the inducer. These temporal selectivity differences of effects can be explained if we consider the nature of cross-modal binding and attention. It has been shown that the temporal synchrony between audio and visual stimuli is the most important cue for cross-modal binding [18], [19]. Cross-modal attentional distraction occurs only when the visual and auditory stimuli are bound together [20], [21]. On the other hand, attentional distraction occurs when one stimulus is presented before the other in a modality by short time. Also, previous studies have indicated that the temporal selectivity of within-modal interaction is sharper than that of cross-modal interaction [22], [23]. Thus, the idea that attentional distraction can modulate the auditory continuity illusion seems to hold for both auditory and visual distracting stimuli.

Effects other than attention distraction may influence the continuity illusion. For example, informational masking, which is often equated with central masking [24], [25], may have affected the continuity illusion: the amount of informational masking of the inducer and the gap was increased by presenting the flanker, and as a result, the continuity illusion was enhanced. In addition, the present findings may be explained by a simpler theory: the default assumption of the auditory system is continuity when one sound is interrupted by another. For the inducee to be perceived as pulsing on and off, there must be sensory evidence for increases in neural activity associated with the onset of the inducee. The flanking tones introduce such evidence, albeit in remote frequency regions. The auditory system may not always be able to judge accurately the frequency region in which transients occur [26]. The present study suggests that the continuity illusion is determined not only by the peripheral auditory system, but also by central attentional factors.

Further research is clearly necessary to establish the involvement of attentional compensation in the continuity illusion; this should involve more direct and systematic manipulation of attention and measures of apparent continuity other than the continuity limit in terms of maximum inducee levels at a fixed inducer levels. Then, the sophisticated mechanisms of the human auditory system that achieve interpretation of any complex auditory scenes would be better revealed.
